# Teachers’ Irrational Belief Scale: Psychometric Properties of the Greek Version and Measurement Invariance across Genders

**DOI:** 10.3390/bs11110160

**Published:** 2021-11-22

**Authors:** Angelos Gkontelos, Julie Vaiopoulou, Dimitrios Stamovlasis

**Affiliations:** 1 Department of Philosophy and Education, Aristotle University of Thessaloniki, 54124 Thessaloniki, Greece; gkontelos@edlit.auth.gr; 2 Department of Education, University of Nicosia, Nicosia 2417, Cyprus; vaiopoulou.cp@unic.ac.cy

**Keywords:** irrational beliefs, psychometric properties, measurement invariance

## Abstract

Irrationality refers to human thoughts and beliefs that signify lack of rationality and entail erroneous perceptions about situational, personal, or collective idiosyncrasies, while it is independent of one’s intellectual ability. Irrational beliefs are ubiquitous in all social and cultural groups and attract a special interest in behavioral sciences, where the primary concern is the development of instruments for identifying and measuring them. The present study evaluates the psychometric properties of Greek version of Teachers’ Irrational Belief Scale (TIBS-G), a 25-item self-reported instrument using data collected from 835 participants. The exploratory procedure, implementing scree plot with parallel analysis, demonstrated the dimensionality of four factors, namely: Self-downing (SD), Authoritarianism (A), Demands for Justice (DJ), and Low Frustration Tolerance (LT). The corresponding reliability measures using Cronbach’s alpha and McDonald’s omega were ranged between 0.70 and 0.80. Subsequently, confirmatory factor analysis showed an adequate fit of the measurement model [*χ*^2^ = 579.98, *df* = 183, *p* < 0.001; *CFI* = 0.960; *TLI* = 0.956; *RMSEA* = 0.051]. In addition, measurement invariance was performed, which demonstrated differences between genders. Finally, discussion on the importance of irrational beliefs and the possible implementation of the TIBS instrument in educational research is provided.

## 1. Introduction

*Irrational beliefs* (IB) are the beliefs adhered to cognitions that signify lack of rationality and often entail misconceptions or erroneous perceptions about situational, personal, or collective idiosyncrasies. The irrational thoughts affect cognitive processes, decision-making and farther human behavior, while they are also associated with emotional states such as frustration and anxiety [1,2]. They are often characterized as *dysfunctional beliefs* or *dysfunctional myths*, which manifest themselves under various situations and in different forms [3]. Researchers have shown a special interest in studying them and have attempted to develop means for identifying and measuring those thoughts. Irrationality, as manifested by human mind is not an easy issue to theoretically define and empirically measure. However, it is omnipresent, and it attracts a considerable attention in many fields. The current work presents a study on the psychometric properties of the Greek version of Teachers’ Irrational Belief Scale (TIBS-G) [4].

### 1.1. Irrationality and REBT

Irrationality is culturally ubiquitous and detected in all social and cultural groups studied historically and anthropologically. Often, human behavior shows evidence of irrational thinking and beliefs, independently of one’s intellectual ability and intelligence. The term of *irrational beliefs* is encountered in many fields of behavioral sciences. Rational-Emotive Behavior Therapy (REBT) [5] defines those beliefs as non-pragmatic, illogical and absolutist, non-reality-based and/or rigid leading to dysfunctional consequences. Conversely, rational beliefs are defined as pragmatic, logical and non-absolutist, reality-based and/or flexible followed by functional consequences [6,7]. In literature, rational beliefs are often characterized as low levels of irrational beliefs. However, research has shown that rational and irrational beliefs are not a simple bipolar construct, but they are distinct dimensions in a more complex structure [8]. Irrational beliefs generate dysfunctional feelings and behaviors because of biasing information processing of specific activating input [9]. In that process, the social-cultural environment has a large share of responsibility since it is the main source of predisposed beliefs of any kind, which could affect individuals [10].

Albert Ellis [5], the REBT’s founder, vehemently states that people, when confronting events which avert their goals, despite their tendencies to use rational thinking, encounter difficulty in dismissing their irrational beliefs that are related to their life’s demands. Irrationality appears resistant to change. In the REBT framework, it is stated that the individuals’ unrealistic and irrational beliefs about themselves or others can provoke unhealthy negative emotions, such as stress, anger and depression [11,12]. In addition, it is pointed out that irrational beliefs bring about not only dysfunctional emotional consequences, but also self-collapsing behaviors, and thus their study is fundamental for understanding human psychological health [13]. To this end, REBT as a cognitive-behavioral approach to psychotherapy had proposed processes-model for confronting related issues [14].

### 1.2. IB’s Dimensions and Their Behavioral Consequences

Ellis’s earlier work describes eleven types of IB [14], however, four main categories prevail: (a) demandingness, (b) self/global downing, (c) awfulizing and (d) low frustration tolerance. These types of IB are associated with various content areas and are relevant to aspects of one’s or other people’s life [15]. Specifically, *demands* are considered to be the root cause of IB, from which all other beliefs come. They play an key role in creating a series of dysfunctional emotions linked with behavioral consequences [13,16]. That is, when a person’s IB relate to self-destruction, the individual evaluates a particular trait, behavior, or action according to his/her desire, and fosters this attitude constantly [17]. Individual *self-downing* show the highest correlation with emotional disorders, affecting anger suppression, along with its violent expressions [18,19]. *Awfulized* IB are referred to the extreme evaluation of a negative event as manipulative [13] and encompass an exaggeration in the description of people or facts as incredibly devastating and destructive [17]. A person possessing such perceptions cannot accept that worse things can happen [20]. Individual awfulized beliefs are associated with anxiety [8], depression, shame, and guilt [21]. Finally, social interactions could not be left unaffected by the individual’s destructive beliefs. Thus, the correlation of these beliefs with the submissive interpersonal attitude of the individual [13], as well as with social isolation [22] is observed. Finally, *low frustration tolerance* discourages people from experiencing unpleasant circumstances [21] and it is associated with inability to suppress anger [23]. These beliefs cause problems of self-control [24], delayed gratification of the individual and attempt self-punishment [25].

As forenamed, IB are defined as evaluative beliefs that are not empirically supported, hindering individuals from achieving their basic aims and causing emotional disruptions [26,27]. Note also that the individual’s evaluations of the beliefs are the cause of the emotional affects and not the situations per se, that people are involved in [6].

### 1.3. Irrationality in the School Framework

The present work focuses on *teachers’ irrational beliefs* (TIB) which relate to educational processes and school practice. TIB refer to intense and rigid in nature thoughts, comprised of extreme beliefs or evaluations. Specifically, irrational beliefs are evaluative cognitions that are identified as the smallest unit of knowledge that can stand as a distinct assertion [28] and are organized in complex schemas representing the person’s constructed concepts of reality, while they affect behavioral responses to that environment [18]. IB definitely appear to be illogical and non-pragmatic, and prominently without empirical support [29].

Although the origin of IB has not been profoundly understood, some speculations based on empirical evidence posit them, as mentioned earlier, within the emotional destruction context. In a career decision-making process, IB or *dysfunctional beliefs* or *myths* are by far influential and both practitioners and researchers attempt to identify and assess them and subsequently discharge or reconstruct them, so that career decision becomes sensible and effective [30]. An environmental stressor has emotional impact on individuals’ beliefs, and especially for teachers, and the unbalance between teaching demands and teaching resources provokes occupational stress. This stress can cause teachers to be susceptible to IB [2]. Indicatively, “Teachers always need a great deal of help from others to solve school-related problems”, “When children have problems, it’s their parent’s fault”, “I must be a perfect teacher and never make mistakes” etc., are examples of listed irrational beliefs specific to teachers [31].

In school practice, some additional demands posited by new situations, such as curriculum innovation, usually intensify TIB, because of the increased fear of failure under the unfamiliar circumstances [32]. Teachers having strong pre-conceived beliefs about a variety of educational issues are challenged by a cognitive dissonance, when the new conditions differ from their initial standard practices. In handling the ensuing dissention, teachers are inclined towards dysfunctional thoughts by fostering overgeneralizations. For instance, when teachers believe “I must effectively instruct this student because I have taught students effectively in the past”, they attempt to rationalize by comparing the present situation with a previous experience. This reasoning adheres obviously to emotional state associated with fear of failure [33].

The conceptual development and theoretical foundation of TIB, as distinct latent constructs, require also to address the measurement issue. The latest developed instrument is the *Teacher Irrational Belief Scale* (TIBS) [4], a self-reporting questionnaire which appraises the different types/dimensions of the TIB. TIB have been examined in several past surveys along with teachers’ stress [2], efficacy beliefs [33], job satisfaction [34], emotional intelligence and psychological hardiness [35], and burnout [36]. Furthermore, dimensional issues are addressed in various populations [37], whereas other researchers have employed the TIBS scale to investigate stress and distress, respectively [2,38].

The TIBS scale entails four dimensions: a) Self-downing, b) Authoritarianism, c) Demands for Justice, and d) Low Frustration Tolerance. More specifically, the *self-downing* subscale refers to high standards that teachers set for themselves, which lead them in overblowing needs for social approval, creating dysfunctional thoughts about reduced personal value because of making mistakes. The *authoritarianism* subscale is associated with teachers’ obstinacy towards their students’ misbehaving. Teachers who adopt authoritarian beliefs support the stern punishment of the students on account of their own disability to cope with students’ behavioral attitudes. The third dimension, *demands for justice*, lays emphasis on teachers’ needs for active involvement in the school functioning. These demands pertain to their concern that they should be listened to and that their collaboration with the administrators be ideal, while they should be considered in decision-making processes etc. Finally, the *low frustration tolerance* dimension indicates the expectations of the teaching difficulties. Many teachers tend to look upon teaching as a complicated procedure, which requires hard work and effort from them.

## 2. Materials and Methods

### 2.1. Participants and Procedures

The survey’s participants (*N* = 839) were educators employed in primary (*n* = 478) and secondary (*n* = 361) education, 75.7% female, with ages varied from 22 to 68 years old (*median* = 45, *mean* = 43.96, *SD* = 10.62). In most cases, their school region was located in a city (*city* = 74%, *town* = 11.8% *country* = 14.2%), while the teaching experience of participating teachers varied from 1 to 39 years (*median* = 15, *mean* = 15.77, *SD* = 10.29) and half of them (57.7%) held a postgraduate degree. The implemented self-completion questionnaire (Teacher Irrational Belief Scale–Greek, TIBS-G) was uploaded on a web-based form via LimeSurvey Forms. Participants were contacted via email to their school email addresses, and responded anonymously at their own time and place. No participants’ responses were excluded from the analyses conducted. Before completion, an accompanying cover letter explained the confidentiality and purpose of the study, the potential objectives, and that the participation was voluntary. No financial incentives were provided for participating in the study.

### 2.2. Instrument

The Greek version, TIBS-G, is an adapted scale from the original TIBS instrument [4], an instrument designed for measuring teachers’ irrational beliefs. The original questionnaire was translated to Greek by the authors followed by an intensive back translation procedure by six bilingual English-Greek speakers who did not know the original English text. The final stage of a collaborative and iterative translation [39] finalized TIBS-G, assuring the proper adaptation, and the questionnaire was used for data collection and the subsequent factor analyses.

For the initial TIBS four dimensions were proposed, namely: *Self-downing* (SD), *Authoritarianism* (A), *Demands for Justice* (DJ), and *Low Frustration Tolerance* (LT). The scale consists of 25 items classified as follows: *Self-downing* {1, 3, 5, 6, 8, 10, 22, 23}, *Authoritarianism* {2, 4, 7, 11, 12, 16, 17, 19}, *Demands* for Justice {13, 14, 18, 21, 25}, *Low Frustration Tolerance* {9, 15, 20, 24}. In responding to the TIBS-G, the participants chose their degree of agreement in a 7-point Likert scale (See Appendix A).

Besides the TIBS-G items, the questionnaire consisted of demographic variables, from which only gender was used in this study for the measurement invariance evaluation.

### 2.3. Analyses

Exploratory Factor Analysis (EFA) by means of Principal Axis Factoring (PAF) was implemented to determine the dimensionality of the TIBS-G, which is expected to conform the four-factor structure. Subsequently, confirmatory factor analysis (CFA) was carried out to test the goodness-of-fit of the measurement model for the theoretically assumed four-dimensional structure. The four-factor model was also compared with a one-factor solution. To evaluate model fit, multiple fit statistics were used: *χ*^2^, comparative fit index (*CFI*), and root mean square error of approximation (*RMSEA*). The accepted values are *CFI* ≥ 0.95, *TLI* ≥ 0.95 and *RMSEA* ≤ 0.05 which indicate a good fit [40]. Some scholars suggest that the values ≤ 0.06 for *RMESA* and *CFI* values ≥ 0.90 could also be acceptable, given that *CFI* values have been shown sensitive to lack of invariance [41,42]. Additionally, the internal consistency of the four scales was examined by calculating Cronbach’s alpha and McDonald’s omega coefficients.

After CFA, the measurement invariance was evaluated following a stepwise procedure, starting with configural invariance, which is the least restrictive model, and then proceeded with more restricted models. In each of the next evaluation steps, the model was compared to the previous one. In this process, every next model was of increasing constraints. The *configurational* invariance, known as pattern invariance, is used as the baseline model. The configurational invariance assures that the factorial structure is the same in the two genders, that is, both male and female share the same goodness of fit statistics, and the same items loads on the same factors. Next, the *metric* invariance is evaluated, which concerns the values of factor loadings in the two groups. If attaining metric invariance, then the construct has the same meaning to participants across sexes. To evaluate the metric invariance, the fit indexes of the metric model are compared to the corresponding configural model, using a *χ*^2^ difference test, where no significance suggests that the factor loadings are invariant across groups, and it advocates that gender-groups share factor variances and covariances. However, it does not account for the comparisons of group means. Then, the *scalar* invariance is evaluated, which, based on metric invariance, tests if the item intercepts are equivalent across the two groups. The item intercepts are considered the starting point/value of the scale, thus, the participants should share the same value if they have the same values on the latent construct. The evaluation of scalar invariance is made by comparing the fit indexes of the scalar model with the corresponding fit of the metric model. If this evaluation resulted in non-invariance of intercepts, then measurement bias might exist, which is suggestive of possible differentiated effects, due to individual differences (between men and women). In other cases, it could be attributed to social or even cultural differences. The last evaluation step is testing for *strict* invariance, which concerns the invariance of factor variances. It includes the overall error in the prediction of the construct, and each variable’s error terms, that is the strict invariance evaluates if the residual errors are equivalent across the two groups.

It is relevant to recall at this point that since the *χ*^2^ difference test is used, in cases of large samples, even very small deviations lead to significant results. For this reason, some scholars have suggested that differences of Δ*CFI* < 0.01 and *ΔRMSEA* < 0.015 should be ignored [41,43].

## 3. Results

### 3.1. Exploratory Factor Analysis (EFA)

**Figure 1:**
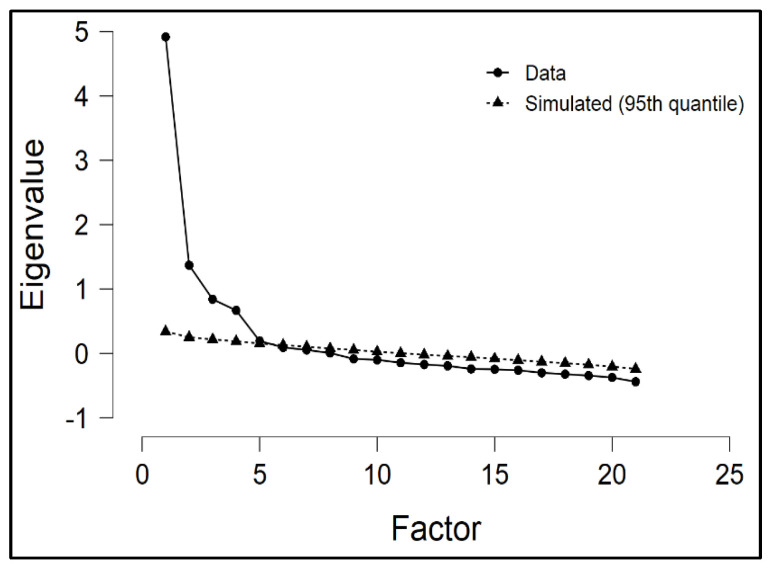
Scree plot with parallel analysis demonstrating the dimensionality of four factors.

In the first step, EFA was applied to the empirical data (*N* = 839) using PAF with Oblique/Promax rotation, to reveal the underlying dimensionality of the TIBS-G scale. Bartlett’s test of sphericity (*χ^2^* = 5126.09, *p* < 0.0001) and the Kaiser–Meyer–Olkin index (0.860) indicated adequate variance for factor analysis. The number of factors was decided taking into consideration the Kaiser’s Criterion (eigenvalue greater than 1), the scree plot and the parallel analysis carried out with simulated data. Figure 1 shows clearly that the factorial structure is four-dimensional.

Note, the first EFA run also showed that four items (SD22, SD23, A2, A19) did not conform to the initially proposed structure demonstrating multiple or low factor loadings (<0.40). These were excluded from the factor model. Table 1 shows the four-dimensional structure, the factor loadings, and the uniqueness of the items, i.e., the variance that is not shared with other variables (the unique variance).

The four factors correspond to *Self-downing* (SD), *Authoritarianism* (A), *Demands for Justice* (DJ) and *Low Frustration Tolerance* (LT), with eigenvalues 2.54, 2.26, 2.01 and 1.74, respectively, while the corresponding portions of variance explained were 12.1%, 10.87%, 9.60%, and 8.30%, respectively.

### 3.2. Reliability Analysis

Reliability measures of the four TIBS-G’s factors were computed using Cronbach’s Alpha (*α*) and McDonald’s omega (*ω*) as following: Self-downing (*α* = 0.777/*ω* = 0.780), Authoritarianism (*α* = 0.779/*ω* = 0.782), Demands for Justice (*α* = 0.754/*ω* = 0.751), and Low Frustration Tolerance (*α* = 0.695/*ω* = 0.704). The overall internal reliability of the TIBS is *α* = 0.848/*ω* = 0.848. These reliability indices suggest that the present measurements with the TIBS-G sub-scales have a satisfactory degree of internal consistency.

### 3.3. Confirmatory Factor Analysis (CFA)–The Measurement Model

Subsequently, CFA was applied to TIBS-G for providing the measurement factor model of TIB. CFA is used to verify the factor structure of a set of observed variables, and it allows testing the hypothesis that a relationship between observed variables and their underlying latent constructs exists. CFA results for the single-factor model are *χ^2^* = 1815.20, *df* = 275, *p* < 0.001, *CFI* = 0.891, *RMSEA* = 0.082, *SRMR* = 0.088, *NFI* = 0.874, however, the proposed four-dimensional model fitted satisfactorily to the empirical data possessing the following fit measure indices: *χ*^2^ = 579.98, *df* = 183, *p* < 0.001; *CFI* = 0.960; *TLI* = 0.956; *RMSEA* = 0.051; *90% CI of RMSEA* = [0.046; 0.056]; *SRMR* = 0.058; *NFI* = 0.942; *GFI* = 0.974. Comparison of the two models by means of a *χ*^2^ test revealed that the four-factor model was substantially improved over the single-factor model (Δ*χ^2^* = 1235.22, *df* = 92, *p* < 0.001). Thus, the hypothesis of the unidimensional structure of TIBS-G for the present data set was rejected. Moreover, no problems with possible model misspecifications were identified when inspecting the standardized residual covariances matrix, since the absolute values of most standardized covariances of residuals were found to be less than two [44]. All calculations were performed using JASP software. Table 2 shows the CFA measurement model and factors, estimates of factor loadings, standards errors, and statistical significance.

Table 3 shows the correlation matrix of the four dimensions, along with the means and the standard deviations of each factor. Self-downing correlated with Authoritarianism (*r =* 0.638, *p* < 0.001) with Demands for Justice (*r =* 0.352, *p* < 0.001) and with Low Frustration Tolerance (*r =* 0.535, *p* < 0.001). Authoritarianism correlates with Demands for Justice (*r =* 0.396, *p* < 0.001) and with Low Frustration Tolerance (*r =* 0.533, *p* < 0.001), while Demands for Justice is correlated with Low Frustration Tolerance (*r* = 0.496, *p* < 0.001).

### 3.4. Measurement Invariance for Gender

Subsequently, the measurement invariance for gender was tested following the procedure described in a previous section. A multi-group CFA model was fitted for each group separately, without any equality constraints. This model tests whether the same factorial structure holds across gender-groups. To evaluate the configural model for each group, the following guidelines for the model fit indices were used: *CFI* > 0.95, *TLI* > 0.95, and *RMSEA* < 0.06 [45]. With sufficient model fit for *configural* invariance, the analysis proceeds to metric invariance evaluation.

In the *metric* invariance model, the factor loadings are assumed to be equal across groups, but the intercepts are allowed to vary between gender-groups. To test metric invariance, a comparison of the configural model against the metric model is made using a chi-square difference (Δ*χ*^2^) test. The *p*-value is statistically significant, thus there is a lack of metric invariance. Even though, due to the non-invariance evidence, the procedure could stop here, all next tests were realized and presented for demonstration purposes. The *scalar* invariance model test is significant, thus, factor loadings and/or intercepts are different in males and females [46]. The *Strict* invariance model test suggests that the residual variances are fixed across groups. Summarizing, in the overall analysis (Table 4), given that the metric and scalar invariance tests are statistically significant, measurement invariance does not hold for gender.

Subsequently, CFA was applied to data separately for the two sexes. The fit indexes of the four-dimensional model for men is {*χ*^2^ = 200.293, *df* = 183, *p* > 0.05; *CFI* = 0.992; *TLI* = 0.990; *RMSEA* = 0.022; 90% CI of *RMSEA* = [0.000; 0.039]; *SRMR* = 0.065; *NNFI* = 0.990; *GFI* = 0.995}, while the fit indexes of the four-dimensional model for women is {*χ*^2^ = 493.57, *df* = 183, *p* < 0.001; *CFI* = 0.961; *TLI* = 0.955; *RMSEA* = 0.052; 90% CI of *RMSEA* = [0.049; 0.057]; *SRMR* = 0.058; *NNFI* = 0.955; *GFI* = 0.996}. Table 5 and Table 6 show the CFA measurement models with factors, estimates of factor loadings, standards errors, and statistical significance, for men and women, respectively.

## 4. Discussion

Behavioral sciences have a strong interest in peoples’ thoughts, attitudes, and beliefs, among which, not surprisingly, irrational beliefs or dysfunctional myths have a great impact on human actions, and they are omnipresent in all societies and cultures. Within educational context, research has shown that irrational beliefs can hinder teachers’ performance, being associated with negative emotions. Teachers possessing those dysfunctional thoughts usually face difficulties understanding students’ feelings and furthermore responding to their delinquent behaviors, while in general, they confront classroom management troubles. Understanding these problems, theoreticians and researchers need to diagnose irrational beliefs, so they can help practitioners to reduce their effects and enhance performance and work interpersonal relationships.

Schools are complex environments, where relationships, co-actions and synergies require a consensus for what is needed to be accomplished and a common understanding about the educational processes. The network of interactions between individual characteristics, which involve the influence of rational and irrational beliefs, is vital behavioral determinants [46], and the investigation of the latter has been a special interest not only for psychiatry, but also for school psychology, rapidly extending to the educational field [2,47,48]. Irrationality becomes a core issue in the contemporary ever-changing society which undergoes rapid changes. Teachers as professionals must support new innovative and demanding situations, going through an essential adaptation, but also via bureaucratic procedures which might be stressful and ineffective. In such sudden changes, given emotional states and the bounded rationality [49], dysfunctional myths are more likely to operate as a defense mechanism against the ensuing uncertainty. TIBS appears a useful tool in research for identifying, understanding, preventing and/or treating those irrational beliefs.

The present study evaluated the psychometric properties of the Greek version of Teachers’ Irrational Belief Scale (TIBS-G). Exploratory and confirmatory procedures revealed the four-factor structure and demonstrated its validity, showing that Self-downing (SD), Authoritarianism (A), Demands for Justice (DJ), and Low Frustration Tolerance (LT) were the four dimensions as initially it has theoretically proposed [4]. In addition, analysis of measurement invariance showed that there are differences between genders and this finding is useful in both measurement procedures and the theoretical consideration. This paper contributes to the measurement issues in the literature of irrational beliefs, by exploring the factorial structure of the TIBS-G, and, thus, further supporting the theoretical framework.

It is imperative to mention at this point that the irrational beliefs or dysfunctional myths are not at all times “dysfunctional”, in the sense that they do not always have negative consequences. As cognitive resources, they are involved in decision making processes and under bounded rationality [49] and play a determinant role. Research has shown that certain types of dysfunctional myths help some people to reach decision easier, while others staying in constant indecisiveness. This unanticipated and peculiar role was explained within complexity theory framework [3,50], where the self-regulation in decision-making is viewed as a nonlinear dynamical process and the ensued taking on as emergent, self-transcending constructions [51], the study of which might illuminate the pathways to rational and irrational human adoptions.

There are of course some limitations of the study which originate from the opportunity sampling procedure. Moreover, measurement invariance, which here is restricted to gender, should be expanded to other categories such as the level of education or teaching specialties. Given that this is the first endeavor appealing to the Greek population, replications of the study should follow to establish firm conclusions.

Conclusively, the present research can support new investigations on human behavior by covering the fundamental requirement in research methodology, that is, the validation of the TIBS-G,. Future inquiries are planned to explore the role of IB within the school context, related to a number of teachers’ individual differences, such as self-efficacy, burnout constructs, creativity, and innovative behaviors.

## Figures and Tables

**Table 1:** Factor loadings of four-dimensional structure.

	Factor 1	Factor 2	Factor 3	Factor 4	
	Self-Downing	Authoritarianism	Demands for Justice	Low Frustration Tolerance	Uniqueness
SD1	0.568				0.735
SD3	0.692				0.570
SD5	0.492				0.757
SD6	0.507				0.514
SD8	0.701				0.477
SD10	0.576				0.542
A4		0.490			0.670
A7		0.519			0.483
A11		0.577			0.677
A12		0.577			0.647
A16		0.760			0.492
A17		0.493			0.532
DJ13			0.512		0.608
DJ14			0.601		0.599
DJ18			0.711		0.528
DJ21			0.669		0.575
DJ25			0.514		0.675
LT9				0.402	0.699
LT15				0.562	0.610
LT20				0.668	0.546
LT24				0.697	0.510

Note. Applied rotation method is promax. Self-downing (SD), Authoritarianism (A), Demands for Justice (DJ), and Low Frustration Tolerance (LT).

**Table 2:** CFA measurement model (4-factor): Factors, estimates of factor loadings, standards errors, and statistical significance.

							95% Confidence Interval		
Factor	Indicator	Symbol	Estimate	Std. Error	z-Value	*p*	Lower	Upper	Std. Est. (All)
SD	SD1	λ11	0.690	0.031	22.33	<0.001	0.629	0.750	0.421
	SD3	λ12	0.862	0.030	29.12	<0.001	0.804	0.920	0.571
	SD5	λ13	0.692	0.031	22.65	<0.001	0.632	0.752	0.426
	SD6	λ14	1.244	0.035	36.01	<0.001	1.176	1.311	0.756
	SD8	λ15	1.202	0.035	34.69	<0.001	1.134	1.270	0.706
	SD10	λ16	1.227	0.036	34.28	<0.001	1.157	1.297	0.702
A	A4	λ21	0.581	0.022	25.98	<0.001	0.537	0.624	0.524
	A7	λ22	1.246	0.034	36.56	<0.001	1.179	1.312	0.775
	A11	λ23	0.695	0.030	23.53	<0.001	0.637	0.752	0.440
	A12	λ24	0.909	0.032	28.29	<0.001	0.846	0.972	0.554
	A16	λ25	0.911	0.031	29.19	<0.001	0.850	0.972	0.580
	A17	λ26	1.105	0.031	35.76	<0.001	1.044	1.166	0.740
DJ	DJ13	λ31	1.248	0.044	28.68	<0.001	1.163	1.334	0.736
	DJ14	λ32	0.942	0.036	26.37	<0.001	0.872	1.012	0.648
	DJ18	λ33	0.795	0.033	24.09	<0.001	0.731	0.860	0.576
	DJ21	λ34	0.654	0.030	22.06	<0.001	0.596	0.713	0.529
	DJ25	λ35	0.737	0.033	22.25	<0.001	0.672	0.802	0.536
LT	LT9	λ41	0.837	0.036	23.26	<0.001	0.767	0.908	0.502
	LT15	λ42	1.033	0.039	26.63	<0.001	0.957	1.109	0.629
	LT20	λ43	1.070	0.040	26.78	<0.001	0.992	1.149	0.632
	LT24	λ44	1.093	0.040	27.43	<0.001	1.015	1.171	0.660

Note: Self-downing (SD), Authoritarianism (A), Demands for Justice (DJ), and Low Frustration Tolerance (LT).

**Table 3:** Factor correlation matrix, means, standard deviations, and internal consistency measures (Alpha, *α* and Omega, *ω*).

	SD	A	DJ	LT
Self-downing (SD)	1.000			
Authoririanism (A)	0.638 ***	1.000		
Demands for Justice (DJ)	0.352 ***	0.396 ***	1.000	
Low Frustration Tolerance (LT)	0.535 ***	0.533 ***	0.496 ***	1.000
Mean Standard Deviation	3.60 (1.13)	3.27 (1.04)	5.45 (1.01)	3.10 (1.20)
Alpha, *α*	0.777	0.779	0.754	0.695
Omega, *ω*	0.780	0.782	0.751	0.704

Note: *** *p* < 0.001.

**Table 4:** Measurement Invariance for Gender.

Invariance Model	*χ* ^2^	*df*	*CFI*	*TLT*	*RMSEA*	*SRMR*	Δ*χ*^2^	Δ*df*	*p*-Value
	0	0							
Configural	693,87	362	0.967	0.961	0.047	0.06	693,9	362	
Metric	750,883	379	0.963	0.959	0.048	0.062	57,01	17	<0.001
scalar	785,491	400	0.961	0.959	0.048	0.064	34,61	21	<0.05
Strict	815,808	421	0.960	0.961	0.047	0.065	30,32	21	<0.10

**Table 5:** CFA measurement model for men: Factors, estimates of factor loadings, standards errors, and statistical significance.

Factor Loadings								
							95% Confidence Interval	
Factor	Indicator	Symbol	Estimate	Std. Error	z-Value	*p*	Lower	Upper
SD	SD1	λ11	0.562	0.063	8.884	<0.001	0.438	0.686
	SD3	λ12	0.823	0.056	14.619	<0.001	0.713	0.933
	SD5	λ13	0.505	0.057	8.837	<0.001	0.393	0.617
	SD6	λ14	1.176	0.069	16.923	<0.001	1.040	1.312
	SD8	λ15	1.001	0.066	15.165	<0.001	0.872	1.131
	SD10	λ16	1.084	0.068	15.839	<0.001	0.950	1.219
A	A4	λ21	0.787	0.058	13.468	<0.001	0.672	0.901
	A7	λ22	1.167	0.068	17.080	<0.001	1.033	1.300
	A11	λ23	0.619	0.056	10.998	<0.001	0.509	0.729
	A12	λ24	0.997	0.074	13.530	<0.001	0.853	1.141
	A16	λ25	0.964	0.069	13.987	<0.001	0.829	1.099
	A17	λ26	0.991	0.063	15.678	<0.001	0.867	1.115
DJ	DJ13	λ31	1.446	0.100	14.415	<0.001	1.250	1.643
	DJ14	λ32	0.849	0.070	12.177	<0.001	0.713	0.986
	DJ18	λ33	0.697	0.064	10.864	<0.001	0.571	0.823
	DJ21	λ34	0.605	0.063	9.624	<0.001	0.482	0.729
	DJ25	λ35	0.682	0.066	10.396	<0.001	0.554	0.811
LT	LT9	λ41	0.987	0.075	13.138	<0.001	0.840	1.135
	LT15	λ42	0.858	0.075	11.469	<0.001	0.712	1.005
	LT20	λ43	1.118	0.086	12.978	<0.001	0.949	1.287
	LT24	λ44	1.135	0.086	13.222	<0.001	0.967	1.303

Note: Self-downing (SD), Authoritarianism (A), Demands for Justice (DJ), and Low Frustration Tolerance (LT).

**Table 6:** CFA measurement model for women: Factors, estimates of factor loadings, standards errors, and statistical significance.

Factor Loadings								
							95% Confidence Interval	
Factor	Indicator	Symbol	Estimate	Std. Error	z-Value	*p*	Lower	Upper
SD	SD1	λ11	0.731	0.035	20.989	<0.001	0.663	0.800
	SD3	λ12	0.870	0.034	25.505	<0.001	0.803	0.937
	SD5	λ13	0.751	0.035	21.314	<0.001	0.682	0.820
	SD6	λ14	1.257	0.039	32.098	<0.001	1.181	1.334
	SD8	λ15	1.263	0.040	31.588	<0.001	1.185	1.341
	SD10	λ16	1.282	0.041	31.163	<0.001	1.201	1.362
A	A4	λ21	0.513	0.022	22.848	<0.001	0.469	0.556
	A7	λ22	1.257	0.038	32.656	<0.001	1.181	1.332
	A11	λ23	0.710	0.034	21.101	<0.001	0.644	0.776
	A12	λ24	0.875	0.035	25.207	<0.001	0.807	0.943
	A16	λ25	0.883	0.034	26.205	<0.001	0.817	0.949
	A17	λ26	1.124	0.035	32.386	<0.001	1.056	1.192
DJ	DJ13	λ31	1.194	0.048	25.002	<0.001	1.101	1.288
	DJ14	λ32	0.973	0.041	23.605	<0.001	0.892	1.054
	DJ18	λ33	0.826	0.038	21.683	<0.001	0.751	0.901
	DJ21	λ34	0.659	0.033	19.877	<0.001	0.594	0.724
	DJ25	λ35	0.755	0.038	19.910	<0.001	0.681	0.829
LT	LT9	λ41	0.796	0.041	19.630	<0.001	0.716	0.875
	LT15	λ42	1.072	0.045	24.017	<0.001	0.984	1.159
	LT20	λ43	1.057	0.045	23.752	<0.001	0.970	1.145
	LT24	λ44	1.079	0.044	24.295	<0.001	0.992	1.166

Note: Self-downing (SD), Authoritarianism (A), Demands for Justice (DJ), and Low Frustration Tolerance (LT).

## Data Availability

The data presented in this study are available on request from the corresponding author.

## Appendix A

**The TIBS-G Instrument**
SD1. To make mistakes or perform poorly as a teacher is for me one of the worst things in the world. SD3. I think I’m really hopeless when I haven’t gotten all my work done on time. SD5. I really should be able to solve all of my students’ problems perfectly. SD6. I can’t stand it when I put all my energy in a student or class and get no results. SD8. I can’t stand being criticized or thought badly of when I haven’t finished something or done it properly. SD10. I think I’m really inadequate when I don’t get approval or respect for what I do. A4. Before I can really change how I feel about a student or class, they must change. A7. I can’t stand it when students misbehave. A11. Students should always be respectful, considerate and behave well. A12. As a teacher, I must have the power to make my students do what I want. A16. Students who constantly misbehave are horrible and should be severely punished. A17. Students can really upset me when they do the wrong things or misbehave. DJ13. I can’t stand it when I am not consulted about a decision, which affects my teaching. DJ14. Without good administrator-teacher communication and support, schools are the very worst and terrible places to work. DJ18. One thing I find totally bad is the lack of communication between teachers and central administration. DJ21. Teachers should be consulted about decisions, which affect their teaching, and it’s really unfair when they are not. DJ25. Schools really should attend more to teachers’ problems, and it is totally unfair when they don’t. LT9. I find it too hard to balance my work and home demands. LT15. School are really lousy places to work because they give teachers too much work and not enough time to do it. LT20. I shouldn’t have to work so hard. LT24. It’s really bad to have to put so many hours both inside and outside the classroom.
